# Editorial: Modeling of cardiovascular systems

**DOI:** 10.3389/fphys.2022.1094146

**Published:** 2022-11-28

**Authors:** Yong Wang, Rupamanjari Majumder, Fang-Bao Tian, Xiang Gao

**Affiliations:** ^1^ Department of Fluid Physics, Pattern Formation and Biocomplexity, Max Planck Institute for Dynamics and Self-Organization, Göttingen, Germany; ^2^ DZHK (German Center for Cardiovascular Research), Partner Site Göttingen, Göttingen, Germany; ^3^ School of Engineering and Information Technology, University of New South Wales Canberra, Canberra, NSW, Australia; ^4^ School of Physics and Information Technology, Shaanxi Normal University, Xi’an, China

**Keywords:** cardiovascular system, modeling, electrophysiology, mechanics, hemodynamics

The mammalian cardiovascular system (CVS) comprises the heart, blood and blood vessels, which, through coordinated action, ensures the transport of oxygen, nutrients, hormones and enzymes to every cell in the body, while collecting toxic wastes for elimination from the same. Because of its complex and multi-component nature, malfunctioning of the CVS often results in multi-scale and multi-physics problems that pose major challenges to targeted therapy (Smith et al., 2004; Walpole et al., 2013; O’Connor et al., 2022). This is because abnormalities occurring at the molecular level can lead to defective electrical or mechanical activities at the organ level (Zhang et al., 2016; Yu et al., 2019), thereby inhibiting the identification of the true source of the problem.

In recent years, mathematical and computational modeling (Smith et al., 2004; Walpole et al., 2013; Niederer et al., 2019) has found synergistic use alongside *in vitro*, *ex vivo* or *in vivo* research, providing useful mechanistic insights through simulations, where experimental capabilities are limited. Inspired by new experimental data and supported by high-performance computing, modeling is also increasingly applied in the CVS to understand mechanisms of abnormal processes and test potential therapeutic strategies (Weinberg et al., 2010; Campbell et al., 2020; Kalhöfer-Köchling et al., 2020; Costabal and Peirlinck, 2021; Pickersgill et al., 2022). To this end, computer models are developed to simulate the state of the art in technology, to guide animal experiments or clinical trials, such as tissue engineering of heart valve (Emmert et al., 2018), atrial defibrillation (Majumder et al., 2021), and aortic stent planning (Ma et al., 2022).

This Frontiers Research Topic focuses on the current state of the art in cardiovascular system modeling, including mathematical or computational aspects, and addresses the fundamental challenges. We are delighted to include 8 articles contributed by 53 authors in this Research Topic. Those articles cover electrophysiology, mechanics, hemodynamics and their coupled effects.

The cardiac muscle is excitable media responsible for the heart contraction under electrical stimulus. The intricate regulation of compartmental Ca^2+^ concentrations in cardiomyocytes is of importance for electrophysiology, excitation-contraction coupling, and complex signaling pathways. Dysregulation of cytosolic Ca^2+^ leads to various pathologies. Streiff and Sachse used a mathematical model of human ventricular cardiomyocyte to investigate the individual contributions of background Ca^2+^ entry and Ca^2+^ leak to the modulation of Ca^2+^ transients and sarcoplasmic reticulum Ca^2+^ loading under static and dynamic conditions. Their results provide quantitative insights into the differential modulation of compartmental Ca^2+^ concentrations by background and leak Ca^2+^ currents, and shed light on the physiological effects of background and leak Ca^2+^ currents and their contribution to the development of diseases caused by Ca^2+^ dysregulations.

In addition to cardiomyocytes, fibroblast are also present in cardiac tissue. Brocklehurst et al. developed a two-dimensional model using the discrete element method and studied the effects of fibroblast-myocyte electrical coupling (FMEC) on atrial electrical conduction and mechanical contractility. Their results show that the coupling slows down the conduction of excitation waves and reduces the tissue strain during contraction. This reveals a role of FMEC in cardiac electrical and mechanical dynamics.

Abnormal propagation of electrical wave in the heart may leads to arrhythmias, such as atrial fibrillation. Patient-specific atrial model, with defined muscle fiber architecture, can be used for risk assessment and treatment planning. Rossi et al. proposed a rule-based definition of fiber orientation in patient-specific left atrium models, and performed electrophysiology simulations. They compared the new algorithm with other rule-based algorithms and demonstrated the robustness and flexibility of the new one.

The cardiac chambers are surrounded by branches of coronary arteries, which deliver oxygen and nutrition to the myocardium. Coronary blood flow is an important indicator in the assessment of coronary artery disease. Munneke et al. proposed a multi-scale model for the coupling between the cardiac mechanics and coronary perfusion, with coronary mechanics and hemodynamics implemented in the closed-loop CircAdapt model. The versatility and validity of the new model was demonstrated in a case study of aortic valve stenosis followed by valve replacement. This model is expected to serve as a platform for studying cardiac-coronary coupling. Using computational fluid dynamics (CFD) modeling, Taylor et al. evaluated the inlet flow rate and microvascular resistance of 27 coronary branches in patients and determined the optimal exponent of Murray’s law (Murray, 1926). The values obtained are lower than the exponent originally proposed by Murray’s law, but are consistent with recent derivations based on theoretical and morphological analyses.

By modeling the coupling between the aortic hemodynamics and mechanics, Zhu et al. investigated the effect of aortic wall compliance on intraluminal hemodynamics within surgically repaired type A aortic dissection. Two patient-specific aortic geometries, either deformable or rigid, were considered. Their CFD results show that the model considering wall compliance is more accurate in predicting wall shear stress, but the model with rigid wall is sufficient to predict pressure drop and computationally cheaper.

Data-driven and machine learning (ML)-based computational models play an important role in understanding cardiac dynamics and hemodynamics. As an application of data-driven technique, Tossas-Betancourt et al. discussed the inconsistencies in routinely acquired anatomical and hemodynamic data from patients with pulmonary arterial hypertension, and proposed and implemented strategies to mitigate these inconsistencies, and then to use this data to inform and calibrate computational models of the ventricles and large arteries. Wang et al. proposed a fast prediction tool for modeling blood flow in stenosed arteries by using a hybrid framework of ML and immersed boundary-lattice Boltzmann method (IB-LBM). Their results show that once the neural network is trained, the prediction of blood flow in stenosed arteries is much more efficient compared to direct CFD simulations.

In summary, this Research Topic contains articles that address different scales, physics, and anatomical components of the cardiovascular system. We believe that these modeling works can increase our understanding of the complex cardiovascular systems and hopefully help physicians to develop new therapeutic strategies. On the other hand, we are aware that in this topic there is a lack of studies that couple all relative physical fields of the heart (Verzicco, 2022), due to the obvious complexity. Based on the current advances in experimental technologies, models that incorporate new experimental data, such as detailed cardiac fiber distribution, are expected. Finally, ML, or ML combined with physical laws (Alber et al., 2019), is expected to have a greater impact in the modeling of the cardiovascular systems.
